# Preclinical evaluation of a live avian metapneumovirus subtype B vaccine strain with cross-protective efficacy in chickens

**DOI:** 10.1016/j.psj.2025.105283

**Published:** 2025-05-10

**Authors:** Sae-Jin Kim, Hyun-Jin Kim, Yun-Hee Noh, Ho-Keun Won, Seung-Min Hong, In-Joong Yoon, Kang-Seuk Choi

**Affiliations:** a Laboratory of Avian Diseases, Research Institute for Veterinary Science and College of Veterinary Medicine for Veterinary Science, Seoul National University, Seoul 088026, Republic of Korea; b Choong Ang Vaccine Laboratories Co., Ltd. (CAVAC), Daejeon 34055, Republic of Korea

**Keywords:** Avian metapneumovirus (aMPV), Subtype B, live vaccine, Cross-protection, Protective efficacy

## Abstract

Avian metapneumovirus (aMPV) infection is an important respiratory disease that causes significant economic loss in the poultry industry. Here, we aimed to develop an effective live aMPV subtype B vaccine strain suitable for mass vaccination of poultry farms to achieve herd immunity. The wild-type virus was attenuated by serial passaging in Vero-K cells, and the genetic stability of the attenuated virus was evaluated through genomic sequencing analysis. The attenuated virus was formulated into test pilot live vaccines. Preclinical tests of the test vaccine, including those measuring indices of safety, minimum immunogenicity, virulence reversion, immunogenicity and cross-protective efficacy, were conducted in SPF chickens. The master seed virus (MSV) of the SNU21004(a) vaccine strain was established by serial passaging in Vero-K cells. Some point mutations occurred throughout the viral genome, but no gene deletions or insertions were observed. The clinical scores, histopathological scores, and viral loads of SPF chickens inoculated with the MSV were significantly lower than those inoculated with the wild-type virus (*P* < 0.05). There was no increase in viral virulence after five backpassages in chickens. The minimal immunogenicity dose for protection against aMPV is 10^2.1^ TCID_50_/dose. The test vaccine induced a strong humoral immune response and efficiently protected chickens, even in vaccinated birds with low antibody titers, regardless of the route of administration (drinking water or spraying) or challenge virus subtype (subtypes A and B). The SNU21004(a) vaccine strain was found to be safe and highly immunogenic even at small doses in chickens and showed cross-protection against other aMPV infections.

## Introduction

Avian metapneumovirus (aMPV) is an important pathogen involved in complicated respiratory disorders in poultry, especially chickens and turkeys. aMPV preferentially replicates in the upper respiratory tract, especially in the nasal turbinate, with short viremia (Jirjis et al., 2002; Aung et al., 2008; Hong et al., 2024). This leads to damage to the ciliary epithelium and results in ciliostasis and deciliation (complete ciliary loss), which abrogates the mucociliary defenses of the upper respiratory tract (Aung et al., 2008; Salles et al., 2023). This results in the main clinical signs of infection in poultry, including sneezing, nasal discharge, rales, frothy eyes and conjunctivitis, and swollen head syndrome (SHS) (Cook, 2000; Salles et al., 2023). Under experimental conditions, chickens present mild respiratory signs, but reproducing swollen head syndrome (SHS) is difficult (Aung et al., 2008; Kwon et al., 2010; Rautenschlein et al., 2011). However, the virus has been frequently associated in the field with SHS, which is usually accompanied by secondary infections that increase mortality in broilers and broiler breeders (Jones et al., 1991; Shin et al., 2000; Choi et al., 2009; Yu et al., 2019; Al-Hasan et al., 2022; Salles et al., 2023).

Metapneumovirus is a member of the *Metapneumovirus* genus in the family *Paramyxoviridae*. The viral genome encodes nine proteins, including nucleoprotein (N), phosphoprotein (P), matrix (M), fusion (F), second matrix (M2-1 and M2-2), small hydrophobic (SH), attachment glycoprotein (G), and large RNA-dependent RNA polymerase (L), in the order of 3′ to 5′ ends (Broor and Bharaj, 2007). Among these, the F and G proteins are the major metapneumovirus surface glycoproteins (Broor and Bharaj, 2007). The F protein is a class 1 viral fusion protein that mediates virus entry and fusion with the host cell membrane and is the primary HMPV antigenic determinant, eliciting protective neutralizing antibodies (Skiadopoulos et al., 2006; Cox and Williams, 2013). The G protein is a predicted type II transmembrane protein that may contribute to attachment *via* interactions with cellular receptors, resulting in weak immunogenicity and significant genetic variability (Juhasz and Easton, 1994; Bäyon-Auboyer et al., 2000; Skiadopoulos et al., 2006; Thammawat et al., 2008).

To date, there are four recognized distinct subtypes of aMPV (A, B, C, and D) with genetic variations and antigenic differences. Subtypes A and B are the major subtypes causing substantial economic losses in poultry, but aMPV-B is more prevalent than aMPV-A in many countries in Europe, Asia, and Africa (Franzo et al., 2020, 2024).

Vaccination is a critical tool for controlling aMPV in poultry, but it must be combined with good biosecurity, proper flock management, and monitoring of vaccine efficacy. There are two main types of commercial vaccines available used to prevent aMPV infection in poultry in the field: live attenuated and inactivated (killed) vaccines. Live attenuated vaccines have been used in young birds to reduce clinical disease incidence and prevent respiratory complications via early immunity, especially protective mucosal immunity in the respiratory tract, and are typically administered via spray, drinking water, or eye drops. Inactivated vaccines administered via injection are usually given to breeders and layers to provide booster and long-term immunity. In the field, the selection of vaccine type, administration route, and vaccination timing is essential to achieve optimal protection in commercial poultry operations. In this study, the attenuated aMPV vaccine strain SNU21004(a) was generated through serial passaging in Vero-K cells and formulated as a test pilot live vaccine. We evaluated the safety, risk of virulence reversion, immunogenicity, and cross-protective efficacy of the vaccine strain to determine whether it could be useful as a live aMPV vaccine in chickens.

## Materials and methods

### Viruses

The virulent field strain aMPV SNU21004(v) belonging to subtype B was kindly supplied by the Laboratory of Avian Disease, College of Veterinary Medicine, Seoul National University (SNU) (Hong et al., 2024). The master seed virus (MSV) of the vaccine strain SNU21004(a) was established by 12 serial passages in Vero E6 cells (ATCC no. CRL-1586) at Seoul National University, followed by plaque purification and 15 serial passages in Vero-K cells, a cloned Vero cell line (ATCC CCL-81), at CAVAC (Daejeon, Korea).

Three virulent aMPV strains, SNU21004(v) (subtype B), K655/07 (subtype A) (Kwon et al., 2010) and SC1509 (subtype B) (Choi et al., 2009), were used as challenge viruses in this study. The challenge viruses were maintained in a chicken embryo tracheal organ culture (TOC) as previously described (Choi et al., 2009; Hartmann et al., 2015). Infectious viruses were quantified via an end-point titration assay using Vero-K cells cultured in 96-well plates according to a previously described protocol (Hong et al., 2024). Viral titers are expressed as the 50 % tissue culture infective dose (TCID_50_) per ml, which was calculated using the Reed and Muench method (Reed and Muench, 1938). All viruses were stored at −80 °C until use.

### Genetic stability test

The MSV of the vaccine strain SNU21004(a) was serially passaged in Vero-K cells as previously described (Hong et al., 2024). The genetic stability of the vaccine strain was evaluated through genome sequence analysis to determine whether mutation events occurred during MSV passage, as previously described (Hong et al., 2024). Viral RNA was extracted from cell culture supernatants containing aMPV using an Allex® DNA/RNA Prep Kit (GeneAll, Korea) according to the manufacturer’s instructions. RT‒PCR was performed using a PrimeScript™ One-Step RT‒PCR Kit (Takara, Japan) according to the manufacturer’s instructions and using previously described PCR primer sets (Hong et al., 2024). The amplified PCR products were purified and sequenced using an ABI 3711 automatic sequencer (Macrogen Co., Seoul, Republic of Korea). The nucleotide sequence data were translated into amino acid sequences, which were subsequently aligned and analyzed using QIAGEN CLC Main Workbench 5 software (QIAGEN, USA).

### Preparation of test vaccine products

The vaccine strain SNU21004(a) was propagated in Vero-K cells and used as a viral antigen for the test pilot vaccines (Table 1). Seven test vaccine products containing various virus concentrations were prepared in a freeze-dried a monovalent formulation in accordance with the CAVAC live vaccine formulation and production protocol and stored in a dark and cool place (2-8°C) until use. Four test vaccine lots, A (L323AMPN01), B (L323AMPN02), C (L323AMPN03) and D (L323AMPN04), were manufactured to determine the optimal virus antigen amount through a minimum immunogenicity test. The remaining three test vaccine lots, E (T323AMPN01), F (T323AMPN02) and G (T323AMPN03), were manufactured on different days to contain the optimal concentration of antigen determined through the minimum immunogenicity test and were used for immunogenicity and protective efficacy tests.

**Table 1:** Test pilot vaccines based on a live attenuated subtype B aMPV used in the study.

Test vaccine	Lot number	Virus strain	Virus titer (per dose)	Purpose
**A**	L323AMPN01	SNU21004(a)	10^1.8^ TCID_50_	Minimum immunogenicity test
**B**	L323AMPN02	SNU21004(a)	10^2.1^ TCID_50_	Minimum immunogenicity test
**C**	L323AMPN03	SNU21004(a)	10^2.4^ TCID_50_	Minimum immunogenicity test
**D**	L323AMPN04	SNU21004(a)	10^2.7^ TCID_50_	Minimum immunogenicity test
**E**	T323AMPN01	SNU21004(a)	> 10^2.1^ TCID_50_	Immunogenicity and efficacy test
**F**	T323AMPN02	SNU21004(a)	> 10^2.1^ TCID_50_	Immunogenicity and efficacy test
**G**	T323AMPN03	SNU21004(a)	> 10^2.1^ TCID_50_	Immunogenicity and efficacy test

### Animal experiments

Specific pathogen-free (SPF) white leghorn chickens (3-4 weeks old) supplied by the CAVAC, were maintained in positive pressure high-efficiency particulate air-filtered stainless steel isolation cabinets (Three Shine, Daejeon, Korea) under constant illumination within a biosafety level-2 laboratory at the CAVAC animal experiment facility. All animal experiments in the study were conducted at the CAVAC Animal Facility under the guidelines established by its Institutional Animal Care and Use Committee (IACUC). All animal procedures performed in this study were reviewed, approved, and supervised by the IACUC of CAVAC (Permit number: 231215-008).

### Animal experiment 1: MSV safety

The safety test of the vaccine strain SNU21004(a) was conducted in SPF chickens receiving a dose of MSV corresponding to 10 doses of vaccine. Briefly, three-week-old SPF chickens (*n* = 10) were inoculated oculonasally with MSV (10^4.1^ TCID_50_ per dose). Clinical signs were observed daily for five days. The severity of the clinical symptoms was scored as previously described (Naylor et al., 2004): 0= no clinical signs, 1= clear nasal exudate, 2= turbid nasal exudate, and 3= frothy eyes or swollen infraorbital sinuses in conjunction with nasal exudate. Five days after inoculation, the birds were humanely euthanized, necropsied, and the nasal turbinates were collected for viral load testing via quantitative RT‒PCR (qRT‒PCR) and histopathological evaluation as previously described (Hong et al., 2024).

### Animal experiment 2: Virulence reversion

A virulence reversion test was performed by serial passaging of the MSV described above. For the virulence reversion test, SPF chickens (*n* = 10) were inoculated oculonasally with nasal turbinate homogenate samples taken from SPF chickens that received MSV. Three days after inoculation (the optimal date was determined through a preliminary study), the birds were humanely euthanized, necropsied, and the nasal turbinates were collected for viral load testing via qRT‒PCR and histopathological evaluation in the same manner described above. Viral passage in SPF chickens was repeated five times in the same manner. At the fifth passage (P5), a negative control group (*n* = 5) receiving PBS alone was also included.

### Animal experiment 3: Minimal immunogenicity

Minimum immunogenicity testing was conducted in three-week-old SPF chickens with test vaccines A (10^1.8^ TCID_50_/dose), B (10^2.1^ TCID_50_/dose), C (10^2.4^ TCID_50_/dose) and D (10^2.7^ TCID_50_/dose). Fifty SPF chickens were divided into four test groups (*n* = 10) and a control group (*n* = 10). Each of the four test groups was vaccinated with the test vaccine via drinking water. Three weeks after vaccination, blood samples were collected from all the groups for serological testing via ELISA, and then all the groups were challenged oculonasally with 10^4.0^ TCID_50_/dose of the wild-type SNU21004(v) strain. Clinical signs were observed daily for five days after challenge, and the severity of clinical symptoms was scored as described above for animal experiment 1. Five days after the challenge, all chickens were humanely euthanized, and nasal turbinate samples were collected for histopathological examination and quantitative RT‒PCR.

### Animal experiment 4: Vaccination route immunogenicity and protective efficacy

The immunogenicities and protective efficacies of the three test vaccines (D, E, and F) in three-week-old SPF chickens were evaluated according to vaccination route. Briefly, 70 SPF chickens were randomly divided into three test groups (*n* = 20) and a control group (*n* = 10). The test groups were divided into two groups (*n* = 10) per vaccine lot; one group was administered the vaccine through drinking water, whereas the other group was vaccinated by coarse spraying (droplet size of 16.8 to 64.3 μm) (Three-Shine Inc., Daejeon, Korea). Blood samples were collected from each chicken 3 weeks after vaccination, and serological tests were performed using ELISA. Four weeks after vaccination, all the groups were challenged oculonasally with the wild-type SNU21004(v) strain (10^4.0^ TCID_50_/dose). Clinical signs were observed twice daily for 5 days after viral challenge, and the severity of clinical symptoms was scored as described above for animal experiment 1. Five days after challenge, the chickens were humanely euthanized, and turbinate samples were collected from each bird for histopathological examination and quantitative qRT‒PCR.

### Animal experiment 5: Cross-protection for subgroup A and B viruses

The cross-protective efficacy of the vaccine strain SNU21004(a) against aMPV subtypes A and B was evaluated in SPF chickens. Briefly, 30 three-week-old SPF chickens were used. Test vaccine E was administered to 20 SPF chickens via drinking water, and 10 SPF chickens were administered PBS in the same manner. Three weeks later, blood samples were collected from all birds immediately before challenge, and the vaccination group was divided into two groups (G-P1 and G-P3). Group P1 was challenged oculonasally with subtype A (K655/07 strain, 10^4.0^ TCID_50_/dose), and group P3 was challenged oculonasally with subtype B (SC1509 strain, 10^4.0^ TCID_50_/dose). The control group was divided into two groups (G-P2 and G-P4). Groups G-P2 and G-P4 were challenged with subtype A aMPV and subtype B aMPV, respectively, in the same manner. Clinical signs were observed twice daily for 5 days after the challenge, and the severity of clinical symptoms was scored as described above for animal experiment 1. Five days after challenge, the chickens were humanely euthanized, and turbinate samples were collected from each individual for histopathological examination and quantitative qRT‒PCR.

### Serological tests

Serum samples were tested for the presence of aMPV antibodies using a quantitative commercial ELISA kit (FlockChek Avian Pneumovirus Antibody, IDEXX Laboratories, Westbrook, ME, USA) according to the manufacturer’s instructions. On the basis of the optical densities of the samples (405 nm), sample-to-positive (S/P) ratios were calculated and converted to ELISA titers according to the calculation formula provided by the manufacturer. When the ELISA titer was 1,655 or higher, the sample was considered antibody positive.

### Histopathology

The tissue samples collected in the study were sent to AviNext Company (Chengju, Korea) for histopathological examination. Briefly, nasal turbinate samples were fixed in 10 % (v/v) buffered neutral formalin, embedded in paraffin, sectioned at 5 μm, mounted on glass slides, and stained with hematoxylin and eosin (H&E) according to standard procedures. All H&E-stained sections were examined by light microscopy. The turbinate epithelia, lamina propria, and lumens were examined histologically. The histopathological lesions on both sides of the nasal turbinate were scored according to the loss of cilia (LOC), flattening of the epithelial cells (FEC), and lymphocytic cell infiltration (LPC) severity (0 = normal, 1 = weak, 2 = moderate, and 3 = severe).

### Quantitative RT‒PCR assay

The viral loads in the collected tissue samples were measured by a qRT‒PCR assay. RNA was extracted directly from nasal turbinate samples via a RNeasy kit (Qiagen, USA) according to the manufacturer’s instructions. The qRT‒PCR assay was performed using a Cepheid SmartCycler RT‒PCR instrument (Cepheid, Sunnyvale, CA) using primers and probes as previously described (Kwon et al., 2010). Samples with threshold cycle (C_t_) values between 26 and 35 were considered to have low concentrations of viral RNA, and those with Ct values lower than 26 were considered to have high concentrations of viral RNA. Ct values greater than 35 were considered negative. Ct values were converted into estimated TCID_50_/mL values for measuring viral loads in test samples, which were determined by a real-time PCR standard curve obtained by linear regression analysis of the threshold cycle (C_t_) value (y-axis) versus the known viral titer as previously described (Hong et al., 2024). CFX Maestro 1.1 (Ver. 4.1.2433.1219) software (Bio-Rad, Hercules, CA) was used to convert the Ct values of the test samples to TCID_50_/mL values.

### Statistical analysis

Statistical analysis was conducted using IBM SPSS Statistics 26 (IBM SPSS Statistics for Windows, version 26.0., IBM Corp, Armonk, NY, USA) and Microsoft Excel. The viral titers, ELISA antibody titers, and correlations between groups were calculated using Microsoft Excel software. Differences between groups were assessed via ANOVA with a Tukey–Kramer multiple comparison test. This was followed by Student’s t-test or Tukey’s honestly significant difference (HSD) test as post hoc analysis. For nonparametric data, the Kruskal‒Wallis test was applied, followed by post hoc analysis via Dunn‒Bonferroni adjustment. A *p* value < 0.05 was considered statistically significant.

## Results

### Genetic stability of the SNU21004(a) vaccine strain

**Fig. 1:**
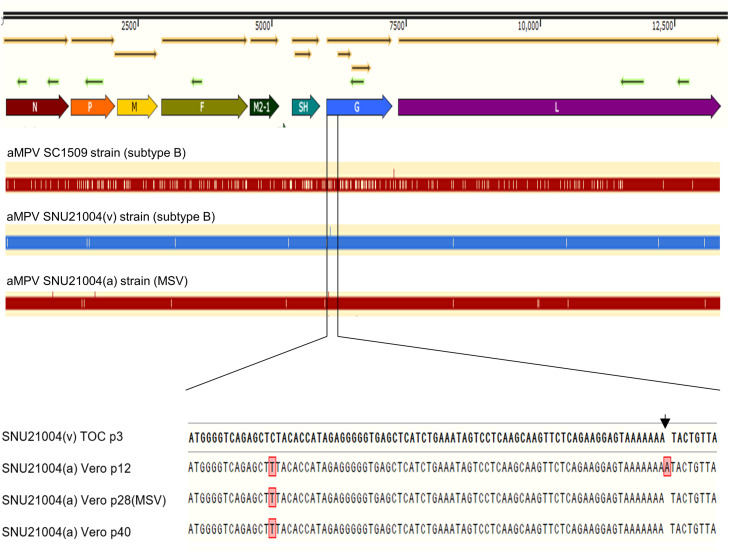
Comparison of the genome sequences of the SNU21004 vaccine strain by serial passaging in Vero cells. The transient A insertion at position 6,098 (located in the N-terminal region of the G protein-encoding gene) found in SNU21004(a) Vero 12 (passage 12) was removed after establishment of the master seed virus (Vero p27). This strain was then maintained for an additional 13 serial passages in Vero-K cells (Vero p40).

The MSV of the SNU21004(a) vaccine strain was serially passaged in Vero-K cells, and the variability of the viral genome was determined through genome sequence analysis, as shown in Fig. 1. When the virulent parent virus, wild-type virus SNU21004(v), was serially passaged to produce an attenuated vaccine virus (MSV), some point mutations occurred throughout the genome, but no gene deletions or insertions were observed. In particular, a short G protein truncated by nucleotide A at position 6,098 of the G gene occurred transiently at the 12th passage in Vero E6 cells (Hong et al., 2024), but the A insertion was removed after selecting the viral clone with an intact G protein-encoding gene through plaque purification. The A insertion at the N-terminal G protein-encoding gene was no longer detected until the final 40 successive passages of MSV in Vero-K cells.

### Evaluation of the attenuation and virulence reversion of the SNU21004(a) vaccine strain in chickens

SPF chickens were inoculated with the MSV (10^4.1^ TCID_50_ per dose) of the vaccine strain SNU21004(a), and clinical symptoms, histopathological changes and upper respiratory tract (nasal turbinate) viral loads were evaluated. The results were compared with those of chickens inoculated with the wild-type virus SNU21004(v) strain (10^4.0^ TCID_50_ per dose) to evaluate attenuation.

**Fig. 2:**
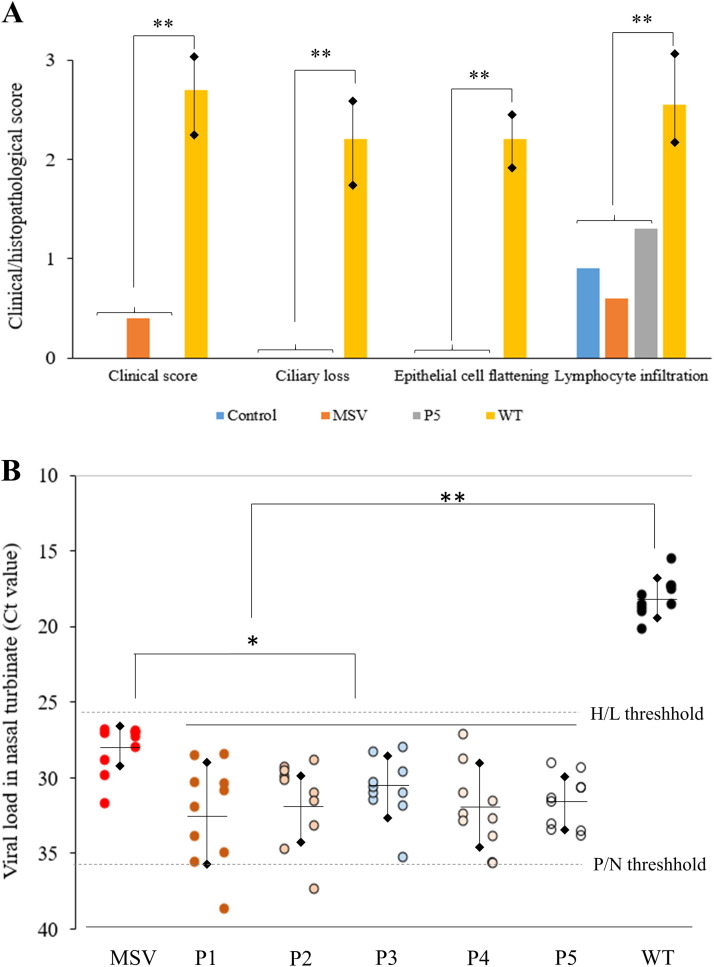
Evaluation of the attenuation and virulence reversion of the SNU21004(a) vaccine strain in chickens. Pathogenicity in chickens was assessed by quantifying clinical signs/histopathological changes (A) and viral shedding according to chicken passage (B) in 3-week-old SPF chickens. Histopathological changes and viral shedding were quantified using nasal turbinate samples collected from birds in each group. (A) MSV, Master seed virus-inoculated group; Px, chicken passaged MSV-inoculated group; WT, wild-type virus-inoculated group. (B) P/N threshold, positive/negative threshold; H/L threshold, high/low viral load threshold.

**Fig. 3:**
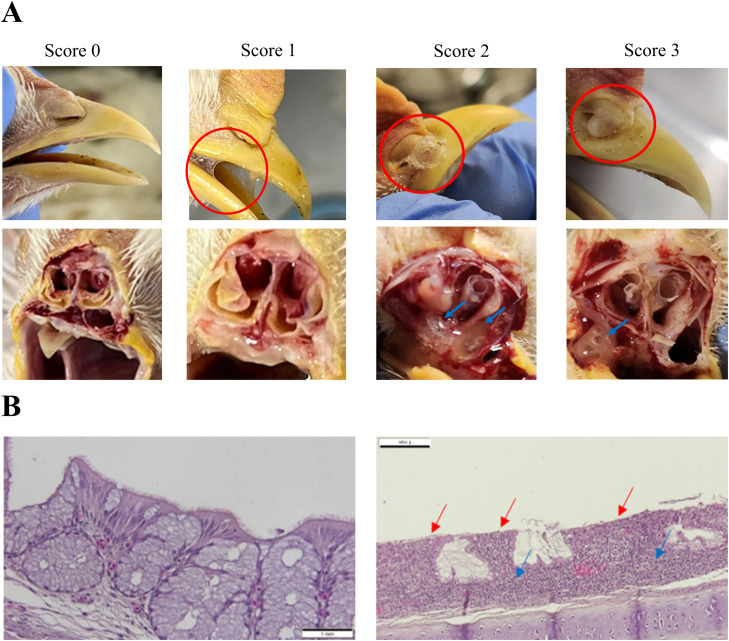
Clinical symptoms (A) and histopathological changes (B) in the nasal cavities of aMPV-infected chickens observed in this study. (A) The severity of clinical signs was scored from 0 (normal) to 3 (infrasinus swelling). Scores of 2 and 3 were observed only in chickens infected with the wild-type virus. (B) Histopathological changes were observed in SPF chickens infected with the wild-type SNU21004(v) strain (right) but not in those infected with the SNU21004(a) vaccine strain (left). Histopathological changes included loss of cilia in the epithelial layer, transformation of the pseudostratified columnar epithelium into a squamous structure (red arrow), and lymphocytic infiltration of the lamina propria (blue arrow) in the nasal turbinates.

The comparative results of the clinical symptoms, histopathological changes and viral loads between the MSV-inoculated group (MSV) and the wild-type virus-inoculated group (WT) are summarized in Fig. 2A. All chickens inoculated with the wild-type virus SNU21004(v) virus presented mild respiratory symptoms, including clear to turbid nasal discharge and infraorbital sinus swelling (mean clinical score of 2.7 ± 0.58) (Fig. 3A). Histopathological changes, including ciliary loss in the epithelial layer (mean score of 2.2 ± 0.42), transformation of the pseudostratified columnar epithelium into a squamous structure (mean score of 2.2 ± 0.35), and lymphocytic infiltration of the lamina propria in the nasal turbinate (mean score of 2.6 ± 0.64), were observed (Fig. 3B). However, in chickens inoculated with MSV, clear nasal discharge was observed in some individuals (3/10), but most were asymptomatic. Accordingly, clinical symptoms (mean clinical score of 0.4 ± 0.52) were significantly lower in the MSV group than in the WT group (*P < 0.05*). Histopathological changes in the nasal turbinates were also significantly reduced compared with those in the group inoculated with the wild-type virus SNU21004(v). Ciliary loss and epithelial cell flattening were not observed in the MSV-inoculated group. Although weak lymphocytic infiltration (mean score 0.6 ± 0.61) was observed in the turbinates, it was significantly lower than that in the WT group (mean score of 2.6 ± 0.64) and not significantly different from that in the mock-infected control group (mean score 0.9 ± 0.74) (*P < 0.05*).

*P < 0.05, ** *P* < 0.001

The viral loads in the nasal turbinates, a preferred tissue for virus replication, were measured via qRT‒PCR, as shown in Fig. 2B. In the WT group, aMPV was detected in the nasal turbinates of all the birds, and the average Ct value was 18.07 ± 1.26 (15.5 to 20.18), which corresponds to an estimated virus titer of 10^5.1±0.31^ TCID_50_/ml. In the MSV group, aMPV was also detected in the nasal turbinates of all birds, but the mean Ct value (28.1 ± 1.63, estimated virus titer 10^2.7±0.40^ TCID_50_/mL) was significantly lower than that in the WT group. The nasal turbinate viral loads did not significantly increase during back passage in chickens (Fig. 2B) (*P < 0.05*). Our results suggest that the vaccine strain SNU21004(a) is highly attenuated compared with the wild-type virus and does not acquire virulence even when back-passaged five times in chickens.

### Immunogenicity and protective efficacy of the vaccine antigen concentration in chickens immunized with the SNU21004(a) strain

A minimum immunogenicity test was performed to determine the minimum antigen dose that can effectively protect chickens from virulent aMPV infection. For this purpose, three-week-old SPF chickens were vaccinated with various titers (10^1.8^ to 10^2.7^ TCID_50_ per dose) of the vaccine strain and challenged with the wild-type SNU21004(v) virus. aMPV-specific antibody levels (three weeks after vaccination) and the level of protection against virulent virus challenge were evaluated.

**Fig. 4:**
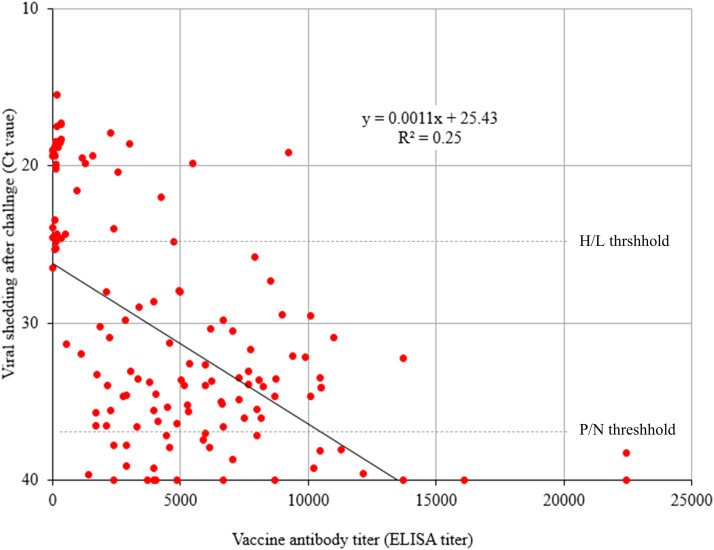
Correlation between vaccine antibody titers before challenge and viral shedding after challenge. Serological and virological data obtained from chickens (*n* = 160) used in this study were used. Scatter plot showing the relationship between the viral load (Ct values) and ELISA antibody titers. P/N threshold, positive/negative threshold; H/L threshold, high/low viral load threshold.

The results are summarized in Table 2. In chickens vaccinated with the test vaccine, the ELISA antibody titers and antibody positivity rates tended to increase as the antigen content increased. The seroconversion rates were 50 % and 90 % in the groups that received test vaccines A (10^1.8^ TCID_50_/dose) and B (10^2.1^ TCID_50_/dose), respectively, and 100 % in the groups that received test vaccines C (10^2.4^ TCID_50_/dose) and D (10^2.7^ TCID_50_/dose). When challenged with wild-type virus after vaccination, chickens in groups G-A2, G-A3, and G-A4, which were vaccinated with test vaccines containing 10^2.1^ TCID_50_/dose or more, did not show clinical symptoms regardless of the vaccination dose given or antibody level measured. In group G-A1, the chickens of which received a vaccination of 10^1.8^ TCID_50_/dose, one chicken presented clear nasal exudate. However, the dose of the vaccine affected the viral loads in the nasal turbinates of vaccinated and challenged birds. In the G-A1 group that received test vaccine A (10^1.8^ TCID_50_/dose), the viral load (mean Ct value of 19.5 ± 1.05, estimated titer of 10^6.75±0.30^ TCID_50_/ml) was high and comparable to that in the nonvaccinated challenge group (mean Ct value of 18.1 ± 1.26, estimated titer of 10^7.15±0.36^ TCID_50_/ml). Among the groups that received test vaccines B, C, and D (10^2.1^ TCID_50_/dose or more), the viral loads in the nasal turbinates were significantly lower than those in the group inoculated with wild-type virus (*P < 0.05*); the mean Ct value was 33.1 ± 2.89 (estimated titer 10^2.19±0.82^ TCID_50_/ml) for group G-A2 (test vaccine B), the mean Ct value was 36.7 ± 4.63 (estimated titer 10^1.89±1.31^ TCID_50_/ml) for group G-A3 (test vaccine C), and the mean Ct value was 34.5 ± 4.84 (estimated titer 10^2.50±1.37^ TCID_50_/ml) for group G-A4 (test vaccine D). However, when the ELISA antibody titer and viral load for each bird were compared, the viral load tended to decrease as the ELISA titer increased, but the correlation between the two values was low (*r* = 0. 48) (Fig. 4). Our results indicate that the vaccine dose required for minimal immunogenicity for protection against aMPV infection is estimated to be 10^2.1^ TCID_50_/dose or higher.

**Table 2:** Immunogenicity and protective efficacy according to vaccine antigen concentration in chickens.

Group^1^	Test vaccine (TCID_50_/dose)	No. birds tested	Vaccine immunogenicity 2		Protection efficacy 3	
			% positive	ELISA titer	Clinical signs	Virus shedding
G-A1	A (10^1.8^)	10	50 (5/10)	2,758 ± 2,704 ^a^	1/10	10/10 (19.49 ± 1.05 ^a^)
G-A2	B (10^2.1^)	10	90 (9/10)	3,601 ± 2,512 ^a^	0/10	6/10 (33.09 ± 2.89 ^b^)
G-A3	C (10^2.4^)	10	100 (10/10)	6,387 ± 2,882 ^b^	0/10	3/10 (36.67 ± 4.63 ^b^)
G-A4	D (10^2.7^)	10	100 (10/10)	7,280 ± 6,310 ^b^	0/10	5/10 (34.53 ± 4.84 ^b^)
Control	PBS	10	0 (0/10)	138 ± 135 ^c^	10/10	10/10 (18.07 ± 1.26 ^c^)

1 Birds were vaccinated via drinking water with each of the test vaccines based on the SNU21004(a) vaccine strain in 3-week-old SPF chickens and challenged oculonasally with 10 ^4^ TCID_50_ per dose of the wild-type SNU21004(v) virus three weeks after vaccination.

2 Vaccine immunogenicity was measured by ELISA in serum samples collected three weeks after vaccination. ELISA titers <1,655 were considered antibody-positive, and the ELISA titers are expressed as the means ± SDs.

3 Clinical signs were observed for 5 days. Viral shedding from the turbinates 5 days after challenge was measured via qRT‒PCR, and Ct values >36 were considered negative. Ct values are expressed as the means ± SDs. Different letters (a, b, and c) represent significant differences (*P<0.05*) between groups within the same column.

### Comparisons of immunogenicity and protection efficacy according to vaccination route in chickens immunized with the SNU21004(a) strain

The effects of the vaccination route on immunogenicity and protective efficacy were evaluated by administering test vaccines E, F, and G via the drinking water (DW) or spray (SP) route to SPF chickens. For each test vaccine, SPF chickens were divided into two subgroups: those receiving vaccination via DW (*n* = 10) and those receiving vaccination via SP (*n* = 10). The ELISA antibody titers (i.e., those induced by vaccination) and protective efficacy (after challenge) results were compared and analyzed according to the test vaccine administered and administration route. The results are summarized in Table 3. When measured by ELISA three weeks after vaccination, seroconversion rates exceeded 90 % in all the DW vaccination groups, and the average ELISA titers were high (mean ELISA titers of 5,536 ± 3,013, 5,167 ± 2,829, and 5,284 ± 2,410). There was no significant difference in vaccine-induced antibody levels between vaccine lots (*P < 0.05*). All the SP vaccination groups presented a seroconversion rate of 100 %, and the average ELISA titers were also high (mean ELISA titers of 5,834 ± 2,675, 5,607 ± 3,108, and 6,094 ± 2,723). There was no significant difference in vaccine-induced antibody levels between vaccine lots (*P < 0.05*). Compared with the DW vaccination group receiving the same vaccine lot, the SP vaccination group tended to have higher average ELISA titers (mean ELISA titers of 5,536 ± 3,013 vs. 5,834 ± 2,675, 5,167 ± 2,829 vs. 5,607 ± 3,108, and 5,284 ± 2,410 vs. 6,094 ± 2,723), but there was no significant difference in ELISA titers between groups vaccinated via different routes (*P < 0.05*).

**Table 3:** Immunogenicity and protective efficacy in chickens immunized with the SNU1004a strain according to vaccination route.

Group 1	Test vaccine (TCID_50_/dose)	Vaccination route	No. birds tested	Vaccine immunogenicity 2		Protection efficacy 3	
				% positive	ELISA titer	Clinical signs	Virus shedding (Ct value)
G1	E (> 10^2.1^)	DW	10	90 (9/10)	5,536 ± 3,013 ^a^	0/10	6/10 (33.1 ± 2.81^a^)
		SP	10	100 (10/10)	5,834 ± 2,675 ^a^	0/10	7/10 (35.7 ± 5.88 ^a^)
G2	F (> 10^2.1^)	DW	10	90 (9/10)	5,167 ± 2,829 ^a^	0/10	5/10 (34.3 ± 6.33 ^a^)
		SP	10	100 (10/10)	5,607 ± 3,108 ^a^	0/10	4/10 (35.2 ± 4.62 ^a^)
G3	G (> 10^2.1^)	DW	10	100 (10/10)	5,284 ± 2,410 ^a^	0/10	6/10 (33.1 ± 5.72 ^a^)
		SP	10	100 (10/10)	6,094 ± 2,723 ^a^	0/10	4/10 (35.7 ± 2.38 ^a^)
Control	PBS	DW	10	0 (0/10)	104 ± 97 ^b^	10/10	10/10 (19.1 ± 0.45 ^b^)

1 Birds were vaccinated via drinking water (DW) or spraying (SP) with each of the test vaccines based on the SNU21004(a) vaccine strain in 3-week-old SPF chickens and challenged oculonasally with 10 ^4^ TCID_50_ per dose of the wild-type SNU21004(v) virus four weeks after vaccination.

2 Vaccine immunogenicity was measured by ELISA in serum samples collected three weeks after vaccination. ELISA titers <1,655 were considered antibody-positive, and the ELISA titers are expressed as the means ± SDs.

3 Clinical signs were observed for 5 days. Viral shedding from the turbinates 5 days after challenge was measured via qRT‒PCR, and Ct values >36 were considered negative. Ct values are expressed as the means ± SDs. Different letters (a and b) represent significant differences (*P<0.05*) between groups within the same column.

No vaccinated groups showed any clinical symptoms during the observation period after challenge with the wild-type virus SNU21004(v), regardless of the route of vaccination and vaccine lot, unlike the control group (unvaccinated and challenged), which presented mild respiratory symptoms such as nasal discharge. However, challenge viruses were detected in both groups at very low or undetectable levels (mean Ct values of 33.10-34.31 for the DW vaccination group and 35.20-35.72 for the SP vaccination group) compared with those of the control group (unvaccinated and challenged) (mean Ct value of 19.1 ± 0.45). Our results indicate that the vaccine strain SNU21004 sufficiently induces protective immunity in SPF chickens and drastically reduces virus shedding after exposure to wild-type aMPV, via either the DW or SP vaccination route.

### Comparisons of the protective effects according to challenge virus subtypes in chickens immunized with the SNU21004(a) strain

We assessed whether the test vaccine had cross-protective efficacy between aMPV subtypes A and B and within aMPV subtype in SPF chickens. As shown in Table 4, the results regarding antibody titers, clinical symptoms, and viral load were compared and analyzed between groups for the test-vaccinated subtype A (K655/07 strain)-challenged group (G-P1), test-vaccinated subtype B (SC1509 strain)-challenged group (G-P3), mock-infected subtype A-challenged group (G-P2) and mock-infected subtype B-challenged group (G-P4), as shown in Table 4. When challenged with subtype A, the G-P1 group (vaccinated and challenged) did not show any respiratory symptoms, whereas the G-P2 group (unvaccinated and challenged) presented mild respiratory symptoms. The results of the subtype B challenge groups were similar to those of the subtype A challenge groups. Group G-P2 (vaccinated and challenged) did not present clinical infections, whereas group G-P4 (unvaccinated and challenged) did. When the viral loads after viral challenge were measured via qRT‒PCR, the virus was detected in the nasal turbinates of all the nonvaccinated groups: G-P2 (mean Ct value of 25.02 ± 1.10) and G-P4 (mean Ct value of 24.42 ± 0.35). However, in the vaccination groups G-P1 and G-P3, challenge viruses were not detected in any of the birds in either group (Table 4). These results suggest that the test vaccine offers intersubtypic and intrasubtypic cross-protection in chickens.

**Table 4:** Cross-protective efficacy in chickens after immunization with the SNU1004(a) strain and postchallenge with virulent aMPV subgroup A or B.

Group 1	Test vaccine (TCID_50_/dose)	No. birds	Challenge Subtype (strain)	Vaccine immunogenicity 2		Protection efficacy 3	
				% positive	ELISA titer	Clinical signs	Viral load (Ct value)
G-P1	E (> 10^2.1^)	10	Subtype A (K655/07)	10/10	7,330 ± 6,302 ^a^	0/10	0/10 (39.11 ± 12.37 ^a^)
G-P2 (control)	PBS	5	Subtype A (K655/07)	0/5	126 ± 134 ^b^	5/5	5/5 (25.02 ± 1.10 ^b^)
G-P3	E (> 10^2.1^)	10	Subtype B (SC1509)	10/10	9,720 ± 4,296 ^a^	0/10	0/10 (37.24 ± 2.71 ^a^)
G-P4 (control)	PBS	5	Subtype B (SC1509)	0/5	155 ± 208 ^b^	5/5	5/5 (24.42 ± 0.35 ^b^)

1 Three-week-old SPF chickens were vaccinated via drinking water (DW) with test vaccine E and challenged oculonasally with 10 ^4^ TCID_50_ per dose of wild-type aMPV subtype A or B three weeks after vaccination.

2 Vaccine immunogenicity was measured by ELISA in serum samples collected three weeks after vaccination. ELISA titers <1,655 were considered antibody positive, and the ELISA titers are expressed as the means ± SDs.

3 Clinical signs were observed for 5 days. Viral shedding from the turbinates 5 days after challenge was measured via qRT‒PCR, and Ct values >36 were considered negative. Ct values are expressed as the means ± SDs. Different letters (a and b) represent significant differences (*P<0.05*) between groups within the same column.

### Correlation between vaccine-induced antibody titers in chickens before challenge and viral shedding after challenge

Many diagnostic laboratories use commercial ELISA kits to monitor aMPV infections and evaluate vaccine programs on poultry farms (Choi et al., 2010; Ball et al., 2022; Conan et al., 2023; Tucciarone et al., 2024). Therefore, we used serological and virological data obtained from the above experiments from chickens (*n* = 160) to analyze the correlation between the ELISA antibody titer immediately before challenge and the viral load (Ct value) measured by qRT‒PCR in the nasal turbinate in the same bird after challenge. The higher the antibody titer induced by the vaccine was, the lower the viral output, but the correlation was not high (R^2^=0.25). In particular, many vaccinated birds shed challenge virus at undetectable or low levels despite low vaccine antibody titers (Fig. 4).

## Discussion

A live attenuated vaccine induces local immunity and subsequent systemic immunity, which provides fundamental immunity to enhance the booster effect of a killed inactivated vaccine. Therefore, the use of commercial vaccines could play an important role in preventing aMPV infections and complex infections in many poultry farms since vaccination prevents complicated infections with other respiratory pathogens through damage to the upper respiratory mucosa. Subtype B aMPVs are the most prevalent aMPVs and cause substantial economic loss in many countries. In this study, we attempted to develop a live subtype B vaccine strain by attenuating the wild-type virus isolated from a farm undergoing an aMPV outbreak in Vero-K cells.

Evaluating the attenuation and protective efficacy of a live attenuated aMPV strain in a chicken model on the basis of only clinical symptoms is very difficult, although SPF chickens show mild respiratory clinical symptoms even in response to virulent viruses (Majó et al., 1995; Ganapathy and Jones, 2007b; Aung et al., 2008; Awad et al., 2015; Youn et al., 2021). The wild-type virus SNU21004(v) used in this study originally caused severe clinical symptoms and death in the field (Hong et al., 2024) but caused mild clinical symptoms such as nasal discharge in experimental SPF chickens. To overcome these limitations, we had to apply a method to measure several parameters quantitatively, including clinical symptoms, viral loads and histopathological changes in the preferred tissue (nasal turbinate).

Compared with the wild-type virus, the vaccine strain used in the present study caused significantly reduced tissue damage in the upper respiratory tract and significantly milder respiratory symptoms. This finding indicates that the vaccine strain SNU21004(a) was highly attenuated, and the attenuation was maintained without virulence reversion during back passages in chickens. These findings suggest that the vaccine strain SNU21004(a) is safe to be used as a live vaccine strain. Virus attenuation was achieved by serial passage *in vitro* in Vero-K cells, as is commonly attempted in live vaccine development (Patnayak et al., 2002; Ganapathy and Jones, 2007b; Sun et al., 2014; Youn et al., 2021). In this study, we identified several point mutations in the G and L protein-encoding genes of the attenuated virus through full-length genetic analysis. However, it is not clear how such point mutations affect pathogenicity. This is why the genetic determinants of viral proteins involved in pathogenicity remain unclear, although there are some reports that the metapneumovirus G protein contributes to pathogenicity to some extent (Govindarajan et al., 2010; Hong et al., 2024). To expand the knowledge of the genetic background of virus attenuation and provide a molecular biological basis for vaccine development, further studies on genetic determinants involved in viral pathogenicity via reverse genetics technology are needed.

The live vaccine strain used in this study induced protective immunity in chickens receiving even a small dose (10^2.1^ TCID_50_). Even in some vaccinated birds, clinical symptoms were effectively prevented despite low vaccine-induced antibody levels. This effect appears to have contributed to the prevention of clinical symptoms not only by reducing the viral load via humoral immunity but also through the involvement of live vaccine virus-induced cellular immunity (Govindarajan et al., 2010; Rubbenstroth and Rautenschlein, 2010; Hartmann et al., 2015; Ballegeer and Saelens, 2020; Lukacs and Malinczak, 2020). These findings suggest that the SNU21004(a) vaccine strain is highly immunogenic and is a strong immune stimulant. If this is the case, our vaccine strain can be useful in the development of bivalent or multivalent vaccines with other live vaccines, such as those for infectious bronchitis and Newcastle disease (Ganapathy et al., 2005, 2006, 2007a; Awad et al., 2015; Lupini et al., 2023). In addition, this vaccine could be used as a vaccine vector for poultry via insertion of a foreign gene (encoding a protective protein for the target pathogen such as avian influenza virus) into a specific site of the genome using reverse genetics technology (Buchholz et al., 2006; Ogonczyk Makowska et al., 2020; Guo et al., 2024).

On commercial poultry farms, mass vaccination with live vaccines to achieve herd immunity is widely used as an important tool for disease prevention and biocontainment (Guerin et al., 2024). Many commercially available live vaccines are mostly administered on farms through drinking water or spray (Youn et al., 2023; Ganapathy et al., 2010; Guerin et al., 2024). In this study, both routes of administration resulted in aMPV antibodies in more than 90 % of the birds, indicating the ability of vaccination via these routes to prevent clinical symptoms and significantly reduce the viral load. These findings suggest that the SNU21004(a) vaccine strain is also suitable for mass vaccination in farm applications. Although aMPV subtypes A and B, which occur mainly on poultry farms, have some antigenic differences, they are known to convey some degree of cross-protection (Ganapathy and Jones, 2007b; Ball et al., 2022; Youn et al., 2023). Our results also demonstrated the cross-protective efficacy of the SNU21004(a) vaccine strain, which efficiently protected against infection with subtype A virus as well as other field viruses of the same subtype. These findings suggest that the SNU21004(a) vaccine strain can be useful for preventing aMPV infection, regardless of the virus subtype circulating in the field. The efficacy of the vaccine strain was evaluated in experimental chickens. There is a need to further evaluate its efficacy as a live vaccine in the future through field trials in the presence of various environmental factors.

## Conclusion

The aMPV strain SNU21004(a) was established as a live vaccine through serial passaging in Vero cells, and its safety, immunogenicity, and protective efficacy were evaluated in SPF chickens. The SNU21004(a) vaccine strain is a highly attenuated virus that causes few respiratory symptoms and little tissue damage in the upper respiratory tract and does not exhibit any tendency to revert or increase in virulence after five back passages in SPF chickens. The vaccine strain was found to be safe and highly immunogenic even at small doses in chickens and showed cross-protective efficacy against aMPV subtype A infection.

## Declaration of competing interest

The authors have no known competing financial interests or personal relationships with other people or organizations that could inappropriately influence their work.
